# Long noncoding RNA* DLEU2* affects the proliferative and invasive ability of colorectal cancer cells

**DOI:** 10.7150/jca.48423

**Published:** 2021-01-01

**Authors:** Xiaoyun He, Bingbing Yu, Gaoyan Kuang, Yongrong Wu, Meili Zhang, Pengfei Cao, Chunlin Ou

**Affiliations:** 1 Department of Pathology, Xiangya Hospital, Central South University, Changsha 410008, Hunan, China.; 2 Department of Pathology, Dezhou People's Hospital, Dezhou 253056, Shandong, China.; 3 Department of Orthopedics, The First Affiliated Hospital of Hunan University of Chinese Medicine, Changsha 410007, Hunan, China.; 4 Department of Hematology, Xiangya hospital, Central South University, Changsha 410008, Hunan, China.; 5 National Clinical Research Center for Geriatric Disorders, Xiangya Hospital, Central South University, Changsha 410008, Hunan, China.

**Keywords:** *DLEU2*, colorectal cancer, invasion, tumorigenesis, survival

## Abstract

Emerging evidence indicates that long noncoding RNAs (lncRNAs) are closely associated with colorectal cancer (CRC) tumorigenesis. One example is lncRNA Deleted in Lymphocytic Leukemia 2 (*DLEU2*). However, how *DLEU2* contributes to CRC is still poorly understood. This study sought to investigate the effects of *DLEU2* on CRC pathogenesis, and the underlying mechanism involved. Using a quantitative real-time polymerase chain reaction (qRT-PCR) assay, we demonstrated that the expression levels of *DLEU2* in 45 pairs of CRC tissues were higher than those in the corresponding normal colon mucosal tissues. In addition, CRC patients with high* DLEU2* expression levels exhibited poor overall survival (OS) and recurrence-free survival (RFS), as determined by analyses and measurements from the GEO and GEPIA databases. When *DLEU2* was silenced using short interfering RNA (siRNA) in CRC cell line, the results demonstrated that *DLEU2* silencing suppressed CRC cell tumorigenesis *in vitro,* which was associated with decreased expression of cyclin dependent kinase 6(CDK6), ZEB1, and ZEB2 as well as enhancing the expression of Cyclin-dependent kinase inhibitor 1A (CDKN1A). Taken together, the results of this study suggested that *DLEU2* may play critical roles in the progression of CRC and may serve as a prognostic biomarker for CRC.

## Introduction

Colorectal cancer (CRC) is the third most commonly diagnosed malignancy and the second leading cause of cancer-related deaths worldwide 1-3. According to epidemiological investigations, in 2012, there were approximately 1.36 million new cases of CRC, and approximately 690,000 deaths, ranking fourth among all malignant tumors 1. With the development of therapeutic approaches, substantial progress has been achieved in treating CRC over the past decades. However, the CRC mortality rate has not changed due to tumor cells metastasis 4,5. CRC progression has been identified as a multistep process that involves inherited and environmental factors 6,7. With the popularity of high-throughput sequencing and molecular therapy, an increasing number of studies have focused on the molecular pathogenesis of CRC, with the aim of exploring suitable biomarkers for identifying the progression and development of CRC 8-10.

Long non-coding RNAs (lncRNAs) are a class of non-coding RNAs (ncRNAs) defined by a transcript length of 200-100,000 nt, and a lack of a complete functional open reading frame (ORF) 11-14. The molecular functions of lncRNAs at the epigenetic, transcriptional, and post-transcriptional levels are conventionally subdivided as follows: 1) acting as host genes for miRNAs; 2) functioning as decoys; 3) acting as a co-regulator or co-repressor; 4) recruiting and interacting with proteins; and 5) interacting with miRNAs. LncRNAs play important roles in the aspects of tissue differentiation and reproduction, individual development, and immunity 11-14. Recent studies have shown that lncRNAs play an important role in various cancers, such as nasopharyngeal carcinoma 15, CRC 16, gastric cancer 17, glioblastoma 18, and cervical cancer 19. These lncRNAs with dysregulated expression may be potential biomarkers valuable in the screening, diagnosis, and therapy of cancers.

In this study, we analyzed two previously published online datasets to analyze the dysregulated expression of lncRNAs in CRC. We found that Deleted in Lymphocytic Leukemia 2 (*DLEU2*) was significantly overexpressed in the two CRC datasets. *DLEU2* is located in the chr13q14.2 region and was originally identified as an important tumor regulator gene 20, whereas little is known concerning the functions and mechanisms of *DLEU2* involvement CRC tumorigenesis. In this study, we showed that *DLEU2* was overexpressed in CRC tissues and cell lines, and high expression levels in CRC patients were associated with poor overall survival (OS) and recurrence-free survival (RFS). Furthermore, *DLEU2* knockdown in CRC cells inhibited malignant proliferation and metastasis. Taken together, these results suggested that *DLEU2* may play a critical role in the progression and development of CRC.

## Materials and Methods

### Tissue samples

A total of 45 CRC lesions and matched adjacent tissues obtained from newly diagnosed CRC patients were collected at the Xiangya Hospital of Central South University. This study was approved by the hospital Research Ethics Board of Xiangya Hospital of Central South University, and all participants provided informed consent for inclusion in the study.

### Bioinformatics analysis

All CRC datasets were deposited in the Gene Expression Omnibus (GEO) database: GSE37364 21, GSE23878 22, GSE41328 23 and GSE17538 24 (these datasets were generated using the Affymetrix Human Genome U133 Plus 2.0 platform). The GSE50760 25 dataset was obtained using the Illumina HiSeq 2000 platform. The GSE37364 dataset has 56 primary CRC samples and 38 normal colon samples, GSE23878 has 35 primary CRC samples and 24 normal colon samples, GSE41328 has 10 pairs of CRC and adjacent non-tumour tissues, and GSE50760 has 18 pairs of metastasis CRC samples and non-metastatic CRC samples.

GSE17538 contains clinical follow-up data for 231 CRC samples. Based on the results of log-rank tests, GSE17538 was divided into a low *DLEU2* expression group (n = 135, i.e. *DLEU2*^low^), and a high *DLEU2* expression group (n = 96, i.e. *DLEU2*^high)^. Subsequently, gene set enrichment analysis (GSEA) 26, 27 was used to identify gene set differences between the two groups.

### Cell culture and transfection

CRC cell lines (SW480, HT29, LoVo, SW620, and CaCO_2_) and human normal colon mucosal cell line (NCM460) were obtained from the American Type Culture Collection (ATCC, Manassas, USA). These cell lines were cultured in RPMI-1640 medium with 10% foetal bovine serum, and HCT116 cells were cultured in Dulbecco's modified Eagle's medium with 10% Tris-buffered saline.

When cell densities reached almost 60%, 50 nM siRNA-*DLEU2* oligo or a negative control (siRNA-NC) were transfected using Lipofectamine 3000 (Invitrogen, USA) according to the manufacturer's instructions. The sequences of the *DLEU2* targeting siRNAs were: siRNA-*DLEU2-1*, 5'- AGUCUACGUUGGAGGUAAA -3'; and siRNA-*DLEU2-2*, 5'- AAGUAUUCAAUCAAAGAAGUG -3'; Sequences of non-target scramble controls were provided by RiboBio (Guangzhou, China).

### Subcellular fractionation analysis

Approximately 1 × 10^7^ HT29 cells were collected in order to determine the cellular localisation of *DLEU2*. Nuclear and cytoplasmic RNAs were collected using a PARIS Kit (Invitrogen). The computing methods employed were described previously 28.

### Quantitative real-time polymerase chain reaction

RNA isolation and amplification and quantitative real-time polymerase chain reaction (qRT-PCR) were performed as described previously 29. The thermocycling program used was as follows: 95°C for 30 sec, followed by 40 cycles of 60°C for 30 sec and 72°C for 30 sec. Primer sequences for qRT-PCR are shown in Table 1.

### Flow cytometry for cell cycle analysis

After transfection with si-NC or si-*DLEU2*, approximately 1 × 10^6^ HT29 cells were collected for flow cytometry assays, and DNA content was detected using propidium iodide (Sigma, San Antonio, USA) staining according to the methods described in a previous study 30. Analysis of cell cycle distribution used cell ModFit software (Beckman Coulter, South Kraemer, USA). Each experiment was repeated three times independently.

### CCK8 assay

Cell proliferation was assayed using the Cell Counting Kit-8 (CCK-8) (Dojin Laboratories, Japan) according to the methods described previously 31. Each experiment was repeated three times independently.

### Western blotting

Cell lysis, electrophoresis, and target protein visualisation were performed according to the methods described previously 32.

### Transwell Matrigel assay

Transwell Matrigel assay were performed using a 24-well transwell plate (8-μM pore size, Costar, America) to detect the invasiveness of CRC cells according to previously described methods 33. Briefly, the number of invasive tumor cells was calculated from the total number of cells from three randomly selected 20× fields for each experiment. The histogram represents data from three independent experiments.

### Statistical analyses

All statistical analyses were performed using GraphPad Prism v.7.0 software and Statistical Package for the Social Sciences version 18.0. Data are shown as mean ± standard error of the mean (SEM), and the results of analysis were considered significant in log-rank tests if *p* < 0.05.

## Results

### DLEU2 is upregulated in CRC

**Figure 1:**
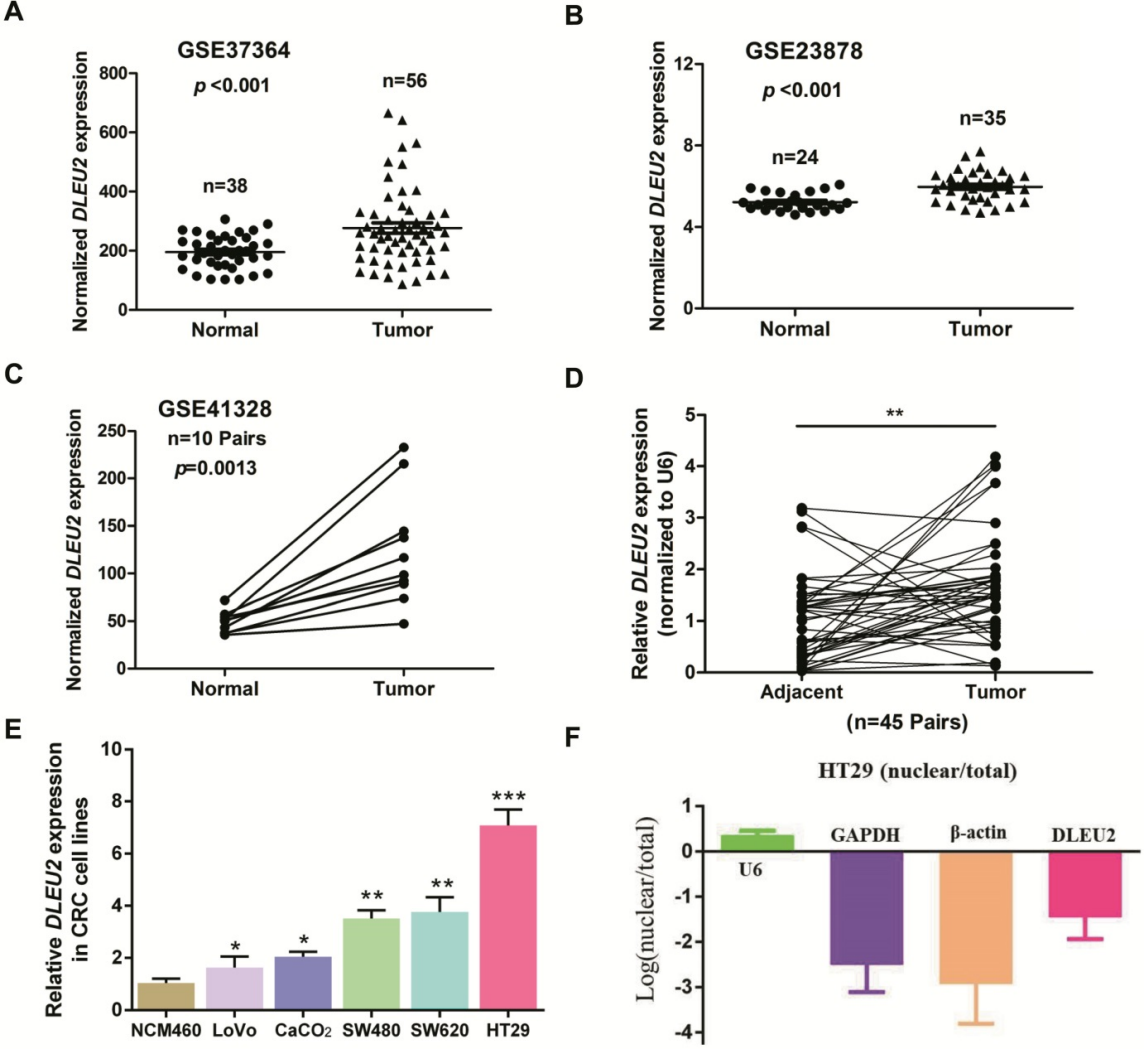
***DLEU2* is upregulated in CRC.** (**A**) #GSE37364 (38 normal colon samples and 56 primary CRC samples), (**B**) #GSE23878 (24 normal colon samples and 35 primary CRC samples) and (**C**) #GSE41328 (containing 10 pairs of CRC tissues and corresponding normal colorectal tissues) identified in the GEO database were used to analyze the expression of *DLEU2*. (**D**) *DLEU2* was highly expressed in the 45 pairs of CRC tissues compared with the corresponding normal colorectal tissues. (**E**) *DLEU2* expression was significantly increased in CRC cell lines (SW480, HT29, LoVo, SW620, and CaCO_2_) compared with NCM460, a normal colon mucosal cell line. (**F**) Nuclear and cytoplasmic RNA were analyzed by qRT-PCR to detect the expression levels of *DLEU2* in HT29 cells. *GPADH* and β-Actin were used as cytoplasmic RNA controls; U6 was used as a nuclear RNA control. Data are shown as means ± SEM. *p < 0.05, **p < 0.01, ***p < 0.001 compared with control.

To identify dysregulated lncRNAs in CRC, we analyzed three online GEO datasets (#GSE37364, GSE23878, and GSE41328) based on the Affymetrix Human Genome U133 Plus 2.0 platform. lncRNA *DLEU2* was significantly upregulated in CRC tissues compared with non-tumor tissues (*p* < 0.05, Fig. 1A-C). Furthermore, we detected *DLEU2* expression in 45 matched pairs of CRC samples and adjacent non-tumor tissues, and our results indicated that *DLEU2* was more highly expressed in CRC tissues than in the adjacent non-tumor tissues (*p* < 0.05, Fig. 1D). *DLEU2* expression levels were also determined by qRT-PCR in five CRC cell lines (SW480, HT29, LoVo, SW620, and CaCO_2_) and in the normal colon mucosal cell line NCM460. *DLEU2* expression was higher in CRC cell lines than in NCM460 cells (all *p* < 0.05, Fig. 1E), and *DLEU2* expression was highest in HT29 cells. Moreover, we analyzed the nuclear and cytoplasmic distribution of *DLEU2* and found that the expression of *DLEU2* was higher in cytoplasm, indicating that the subcellular localization of *DLEU2* in CRC cells was primarily cytoplasmic (Fig. 1F).

### Association between DLEU2 expression and clinicopathological features of CRC

**Figure 2:**
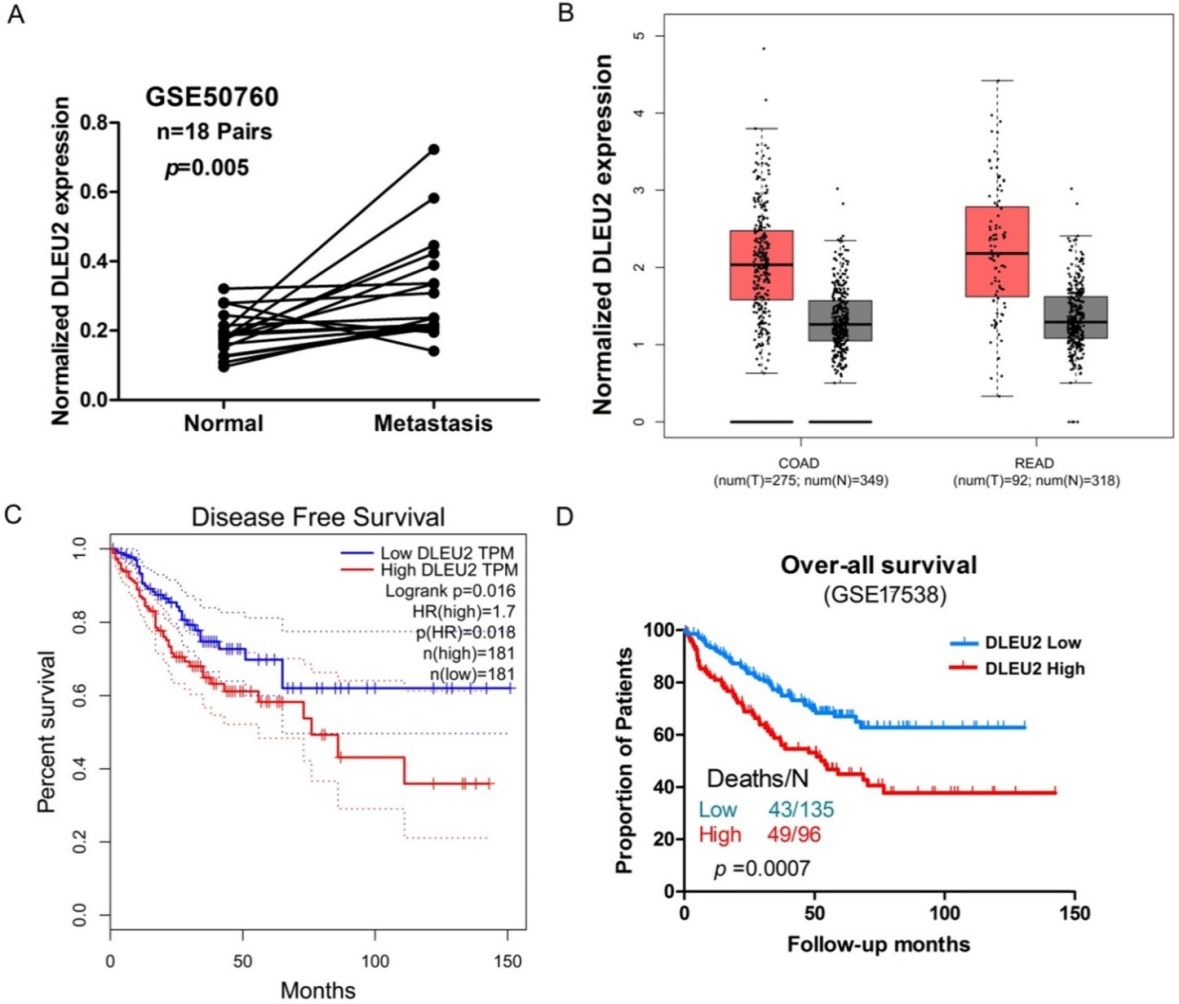
Association between *DLEU2* expression and clinicopathological features of CRC. (A) Relative expression of *DLEU2* in normal and metastatic CRC tissues was obtained from the GEO database (#GSE50760, 18 matched metastatic CRC samples and adjacent non-metastatic samples). (B) The GEPIA database was used to analyze *DLEU2* expression in colon adenocarcinoma (COAD) and rectal adenocarcinoma (READ) samples. (C) The GEPIA database was used to analyze the clinic impact of* DLEU2* expression patterns on CRC patient recurrence-free survival (RFS). (D) Kaplan-Meier analysis showing overall survival (OS) curves for CRC patients with different expression levels of *DLEU2*; statistical significance was assessed by log-rank tests (#GSE17538, the specimen was divided into two groups: group 1, *DLEU2*^low^, n = 135; group 2, *DLEU2*^high^, n = 96).

We next assessed potential correlations involving *DLEU2* expression with clinicopathological features in CRC. We assessed the correlation between *DLEU2* expression and distant metastasis in CRC tissues by analyzing a previously published Affymetrix HG_U133 Plus 2 array dataset (#GSE50760). Elevated expression of *DLEU2* was significantly correlated with CRC distant metastasis (*p* = 0.004, Fig. 2A). Next, by analyzing public CRC datasets in GEPIA 34, we found that *DLEU2* was significantly upregulated in colon adenocarcinoma (COAD) and rectal adenocarcinoma (READ) samples from TCGA data (*p* < 0.05, Fig. 2B; Supplemental Fig. 1), and that high* DLEU2* expression was associated with poor RFS (*p* < 0.05, Fig. 2C). We also examined the association between *DLEU2* expression levels and OS in the GSE17538 database using Kaplan-Meier analysis with log-rank tests. The results revealed that patients with high *DLEU2* expression levels had lower OS (*p* < 0.05, Fig. 2D). Taken together, these data indicated that high *DLEU2* expression is an independent risk factor for CRC patients.

### Knockdown of DLEU2 expression in CRC cells inhibits proliferative and metastatic phenotypes

**Figure 3:**
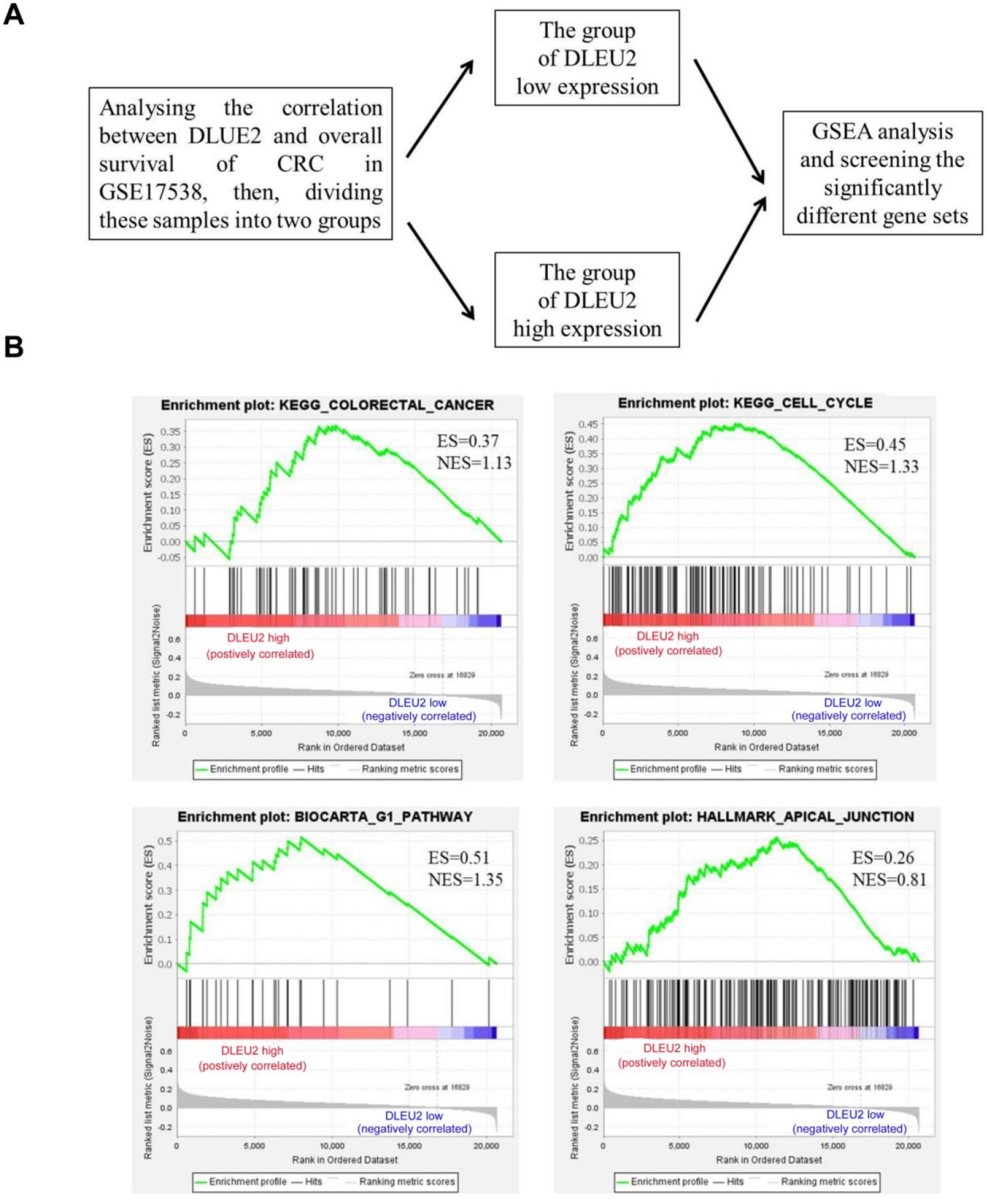
The significantly-different gene sets between CRC specimens with high DLEU2 expression (*DLEU2*^high^) and low DLEU2 expression (*DLEU2*^low^) were analyzed. (A) Schematic flowchart illustrating the strategy to analyze gene set differences between *DLEU2*^high^ and *DLEU2*^low^ cells in CRC specimens from the GSE17538 database revealed by gene set enrichment analysis (GSEA). (B) GSEA identified gene set differences between *DLEU2*^high^ and *DLEU2*^low^. ES: enrichment score; NES: normalized enrichment score.

To explore the function of *DLEU2* in CRC cells, GSEA was used to analyze the significantly-different gene sets between CRC specimens with high *DLEU2* expression (*DLEU2*^high^) and low *DLEU2* expression (*DLEU2*^low^) (Fig. 3A). High expression of *DLEU2* was positively correlated with colorectal cancer, cell cycle, G1 pathway, and apical junction signaling sets (Fig. 3B-E; Supplemental Fig. 2-5).

**Figure 4:**
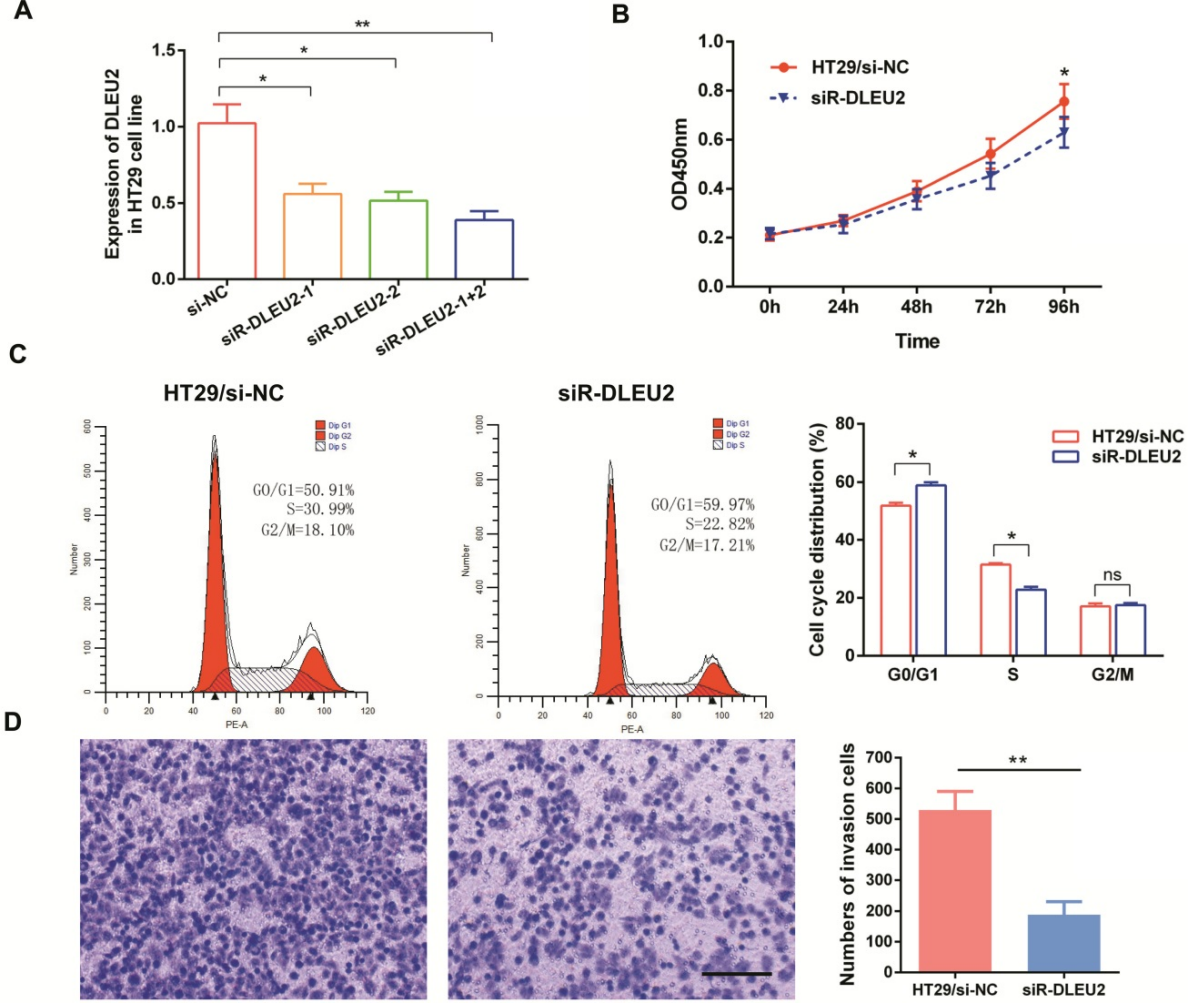
Knockdown of *DLEU2* expression in CRC cells inhibits proliferative and metastatic phenotypes*.* (A) After transfection of HT29 cells with either si-NC or siR-*DLEU2* (1#, 2#, 1+2#) for 48 h, the expression of *DLEU2* was analyzed by qRT-PCR. CCK8 assays (B) and flow cytometry (C) were used to detect cell proliferative ability, and transwell matrigel assays (D) were used to detect cell metastatic ability. Data are shown as means ± SEM. *p < 0.05, **p < 0.01 compared with control.

We also measured the efficiency of short interfering RNA (siRNA) siR*-DLEU2*. *DLEU2* expression in both siRNA siR*-DLEU2-1 + 2* groups was significantly down-regulated compared to siR-*DLEU2-1* and siR-*DLEU2-2* groups in HT29 cells (Fig. 4A). After establishing siRNA efficacy, we assessed the effects of *DLEU2* knockdown in CRC cells. We found that knocking-down *DLEU2* expression significantly inhibited HT29 cell proliferation relative to control cells in 96h (*p* < 0.05, Fig. 4B). Flow cytometric analysis showed that knocking-down *DLEU2* expression in HT29 cells increased the percentage of cells in G1 phase, and decreased the percentage of cells in S phase (*p* < 0.05, Fig. 4C). Subsequently, we explored the effects of *DLEU2* knockdown on the invasiveness of CRC cells using transwell matrigel assays. Knocking-down *DLEU2* expression significantly inhibited the invasive capacity of HT29 cells compared to control group cells (*p* < 0.05, Fig. 4D). These findings suggested that *DLEU2* plays a significant role in CRC tumorigenesis.

### Knockdown of DLEU2 expression in CRC cells influences the expression of proliferation- and EMT-related genes

**Figure 5:**
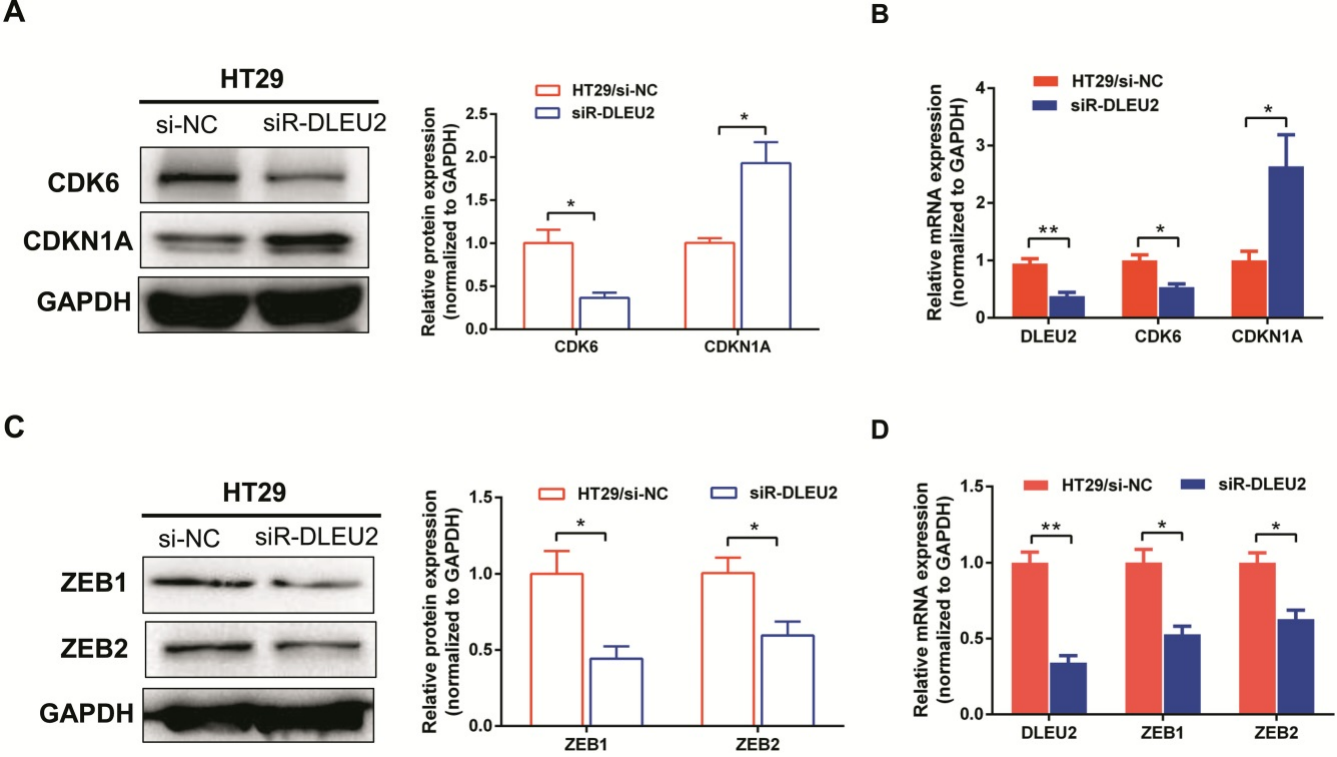
Knockdown of *DLEU2* expression suppresses the expression of proliferation and metastatisis markers in CRC. After transfection with si-NC or siR-* DLEU2* for 48 h, (A-B) mRNA and protein expression levels of cyclin dependent kinase 6 (CDK6) and cyclin-dependent kinase inhibitor 1A (CDKN1A) in HT29 cells were analyzed by qRT-PCR, western blotting and densitometry; (C-D) the mRNA and protein expressionof ZEB1 and ZEB2 in HT29 cells were analyzed by qRT-PCR, western blotting and densitometry. Data are shown as means ± SEM. *p < 0.05, **p < 0.01 compared with controls.

**Figure 6:**
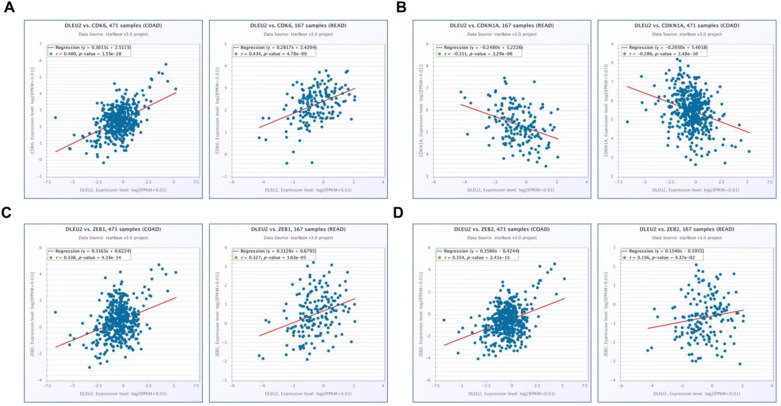
Scatter plots illustrating the statistical association between *DLEU2* expression and levels of proliferative and metastatic markers in CRC*.* The Starbase database was used to analyze associations involving *DLEU2* expression and the expression of CDK6 (A), CDKN1A (B), ZEB1 (C), and ZEB2 (D) in COAD and READ samples.

To further elucidate the molecular mechanisms by which knockdown of *DLEU2* expression suppressed proliferation and invasion by CRC cells *in vitro*, we used qRT-PCR and western blotting to assess mRNA and protein levels of the proliferation markers cyclin‑dependent kinase 6 (CDK6) and cyclin-dependent kinase inhibitor 1A (CDKN1A), as well as the epithelial-mesenchymal transition (EMT) markers ZEB1 and ZEB2 in HT29 cells. Knocking-down *DLEU2* expression significantly inhibited expression of CDK6, and promoted the expression of CDKN1A (*p* < 0.05, Fig. 5A-B). Meanwhile, *DLEU2* knock-down significantly decreased the expression of the EMT markers ZEB1 and ZEB2 (*p* < 0.05, Fig. 5C-D). Starbase database (http://starbase.sysu.edu.cn/) analysis showed that *DLEU2* expression was positively associated with expression of CDK6, ZEB1, and ZEB2, and negatively associated with expression of CDKN1A in COAD and READ samples (all *p* < 0.05, Fig. 6). These results indicated that *DLEU2* may contribute to the regulation of proliferative and EMT marker expression in CRC cells.

## Discussion

Evidence from recent studies has demonstrated that lncRNAs regulate gene expression at the epigenetic, transcriptional and posttranscriptional levels 12, 35. LncRNAs are also involved in the pathogenesis of many diseases, especially cancer. Disruption of lncRNA levels is closely correlated with cancer cell proliferation and apoptosis, EMT, and drug resistance 36, 37. More and more lncRNAs have been reported as being differentially-expressed in CRC and with correlations involving poor prognosis, including *MALAT1* 38, *LINC01234* 39, *AFAP1-AS1* 40, *GAS5* 41, *LINC00152* 42, *NEAT1* 43, among others. lncRNAs are considered ideal biomarkers for tumor diagnosis and monitoring because they can be detected with high specificity and sensitivity, are easy to extract, and are stable in blood and tissues 44.

With the popularity of high-throughput sequencing and RNA-Seq gene microarray technologies, many public databases (for example TCGA, GEO, and Oncomine) have emerged as powerful tools to be used to predict and analyze potentially valuable lncRNAs 45. In this study, we first analyzed dysregulated lncRNAs in CRC using three GEO datasets (#GSE37364, GSE41328, and GSE23878), and found that lncRNA Deleted in Lymphocytic Leukemia 2 (*DLEU2*) was significantly overexpressed in two CRC datasets. *DLEU2*, located in the chr13q14.2 region, was first reported in lymphocytic leukemia 46. Recent studies have shown that *DLEU2* is upregulated in many human cancers, such as non-small cell lung cancer (NSCLC) 47, esophageal adenocarcinoma 48, and osteosarcoma 49. However, the roles and the mechanisms involving *DLEU2* in CRC have rarely been reported. In this study, we found that overexpression of *DLEU2* in CRC patients was associated with poorer RFS and OS, independently of other factors by multivariate analysis.

Recent studies suggest that *DLEU2* plays a critical role in the development and progression of human cancers. Xu et al. 50 demonstrated that overexpressed *DLEU2* can bind with miR-455 to promote the expression of SMAD2, thereby inducing the proliferation and invasion of pancreatic cancer (PC). Moreover, Guo et al. 51 reported that overexpression of *DLEU2* can promote tumorigenesis in hepatocellular carcinoma (HCC) by interacting with the zeste 2 polycomb repressive complex 2 subunit (EZH2). However, the effects of *DLEU2* on the tumorigenesis of other malignancies remain far from being understood*.* In this study, we first identified that the expression of* DLEU2* in CRC was positively correlated with colorectal cancer, cell cycle, G1 pathway, and Apical junction signaling sets by GSEA. We demonstrated that knock-down of *DLEU2* expression in CRC cells could suppress their proliferative and invasive capabilities by reducing the expression of CDK6, ZEB1, and ZEB2, as well as enhancing the expression of CDKN1A. Furthermore, we showed that the subcellular localization of *DLEU2* in CRC cells was mainly cytoplasmic. The subcellular localization of lncRNAs is usually related to their biological functions 52. lncRNAs, mainly localized in the cytoplasm, can 'sponge' miRNAs to form ceRNA so as to regulate the expression of target genes; whereas nuclear lncRNAs can interact with transcription factors or epigenetic modification-associated proteins to modulate gene expression. Therefore, we speculated that *DLEU2* expressed in the cytoplasm of CRC cells may sponge miRNA to form ceRNA, thereby regulating the malignant biological behavior of CRC. In our next study, we will further explore regulatory mechanisms involving *DLEU2* in CRC progression.

In summary, our study indicated upregulated expression of lncRNA* DLEU2* in CRC, and high *DLEU2* expression was positively correlated with poor survival time (RFS and OS) of CRC patients. We also demonstrated that knock-down of *DLEU2* could inhibit the proliferative and invasive capabilities of CRC cells. However, there were certain limitations to this study: 1) we did not further investigate the underlying regulatory mechanisms involving *DLEU2* in promoting the proliferative and invasive capabilities of CRC cells; 2) our study did not extend to animal experiments to explore the effects of *DLEU2 in vivo*. Taken together, our study may provide novel targets for candidate CRC therapies in the future.

## Supplementary Material

Supplementary figures.

## Figures and Tables

**Table 1:** Primer sequence for real-time PCR

Gene	Primer (Forward)	Primer (Reverse)
*DLEU2*	TCCGAGAGTATAGCGCCACT	ACTGCCCTTTGCTCCAAGTA
*CDK6*	GCTGACCAGCAGTACGAATG	GCACACATCAAACAACCTGACC
*CDKN1A*	CGATGGAACTTCGACTTTGTCA	GCACAAGGGTACAAGACAGTG
*ZEB1*	GATGATGAATGCGAGTCAGATGC	ACAGCAGTGTCTTGTTGTTGT
*ZEB2*	GCGATGGTCATGCAGTCAG	CAGGTGGCAGGTCATTTTCTT
*β-actin*	TCACCAACTGGGACGACATG	GTCACCGGAGTCCATCACGAT
*U6*	CTCGCTTCGGCAGCACA	AACGCTTCACGAATTTGCGT
*GAPDH*	AACGGATTTGGTCGTATTGG	TTGATTTTGGAGGGATCTCG
