# Supplementary material for: Long noncoding RNA DLEU2 affects the proliferative and invasive ability of colorectal cancer cells
Source: J Cancer. 2021 Jan 1;12(2):428–37. doi: 10.7150/jca.48423 (PMC7738996; doi:10.7150/jca.48423)
Supplement: Supplementary file 1 — Supplementary figures. [file jcav12p0428s1.pdf]

**Supplementary Figures**

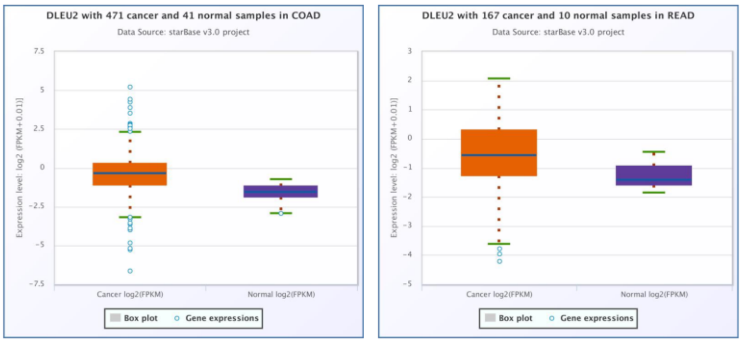


**Supplemental Fig. 1.** The Starbase database was used to analyze the *DLEU2* expression in CRC samples. COAD stands for colon adenocarcinoma and READ for rectal adenocarcinoma.

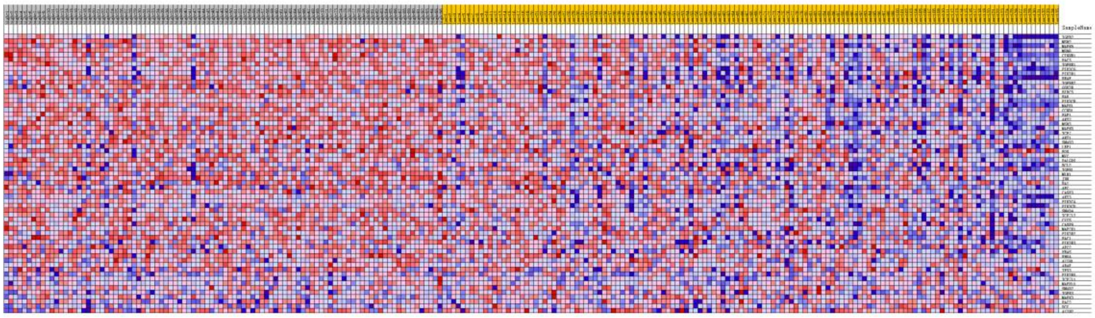

**Supplemental Fig. 2.** The heatmap showed the different genes of "colorectal cancer" gene set between DLEU2$^{low}$ and DLEU2$^{high}$ groups in GSE17538 dataset by the GSEA analysis.

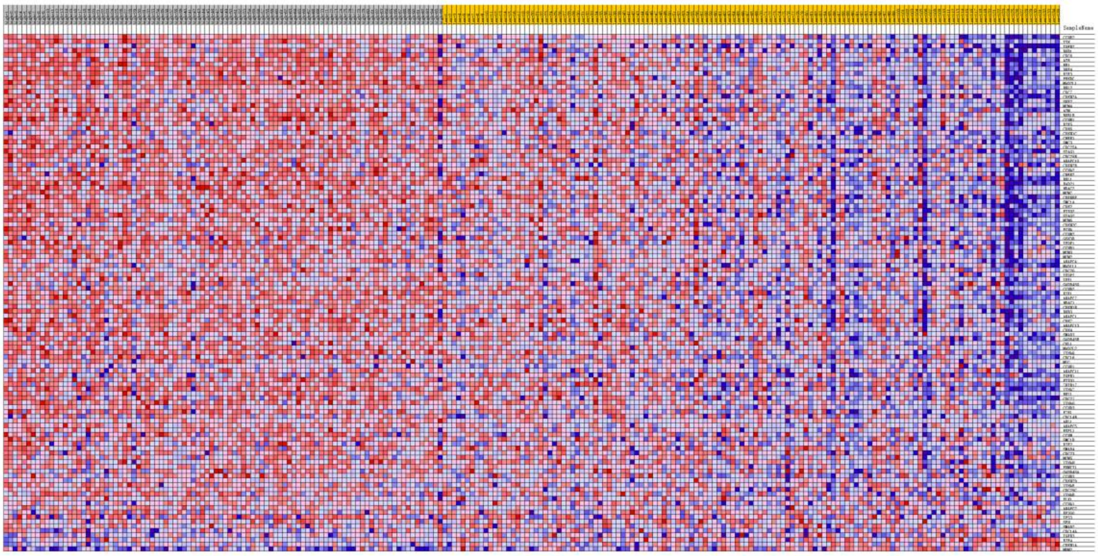

**Supplemental Fig. 3.** The heatmap showed the different genes of "cell cycle" gene set between DLEU2$^{low}$ and DLEU2$^{high}$ groups in GSE17538 dataset by the GSEA analysis.

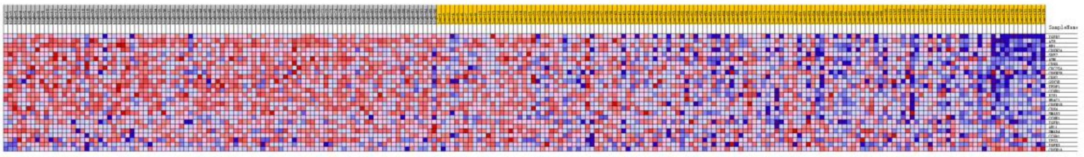

**Supplemental Fig. 4.** The heatmap showed the different genes of “G1 pathway” gene set between DLEU2$^{low}$ and DLEU2$^{high}$ groups in GSE17538 dataset by the GSEA analysis.

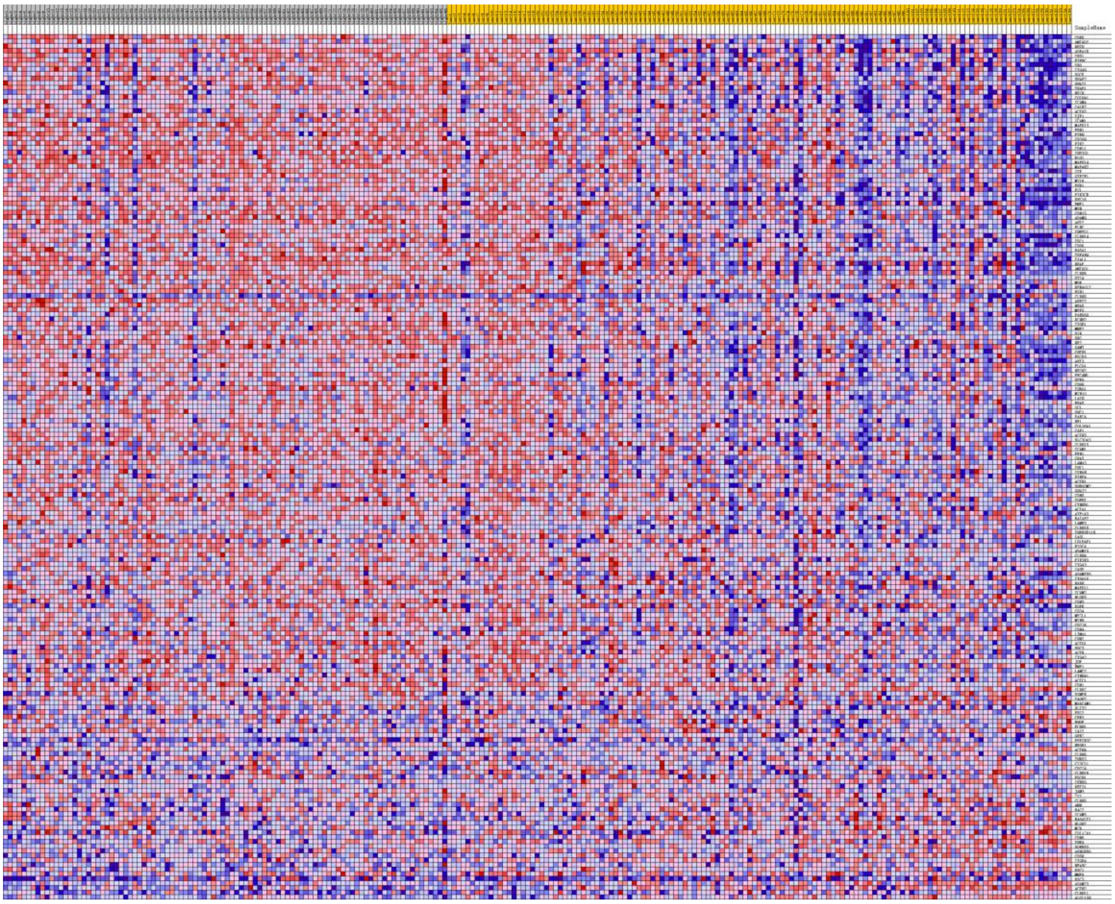

**Supplemental Fig. 5.** The heatmap showed the different genes of “Apical junction signaling” gene set between DLEU2$^{low}$ and DLEU2$^{high}$ groups in GSE17538 dataset by the GSEA analysis.
